# NGF reprograms metastatic melanoma to a bipotent glial-melanocyte neural crest-like precursor

**DOI:** 10.1242/bio.030817

**Published:** 2017-11-24

**Authors:** Jennifer C. Kasemeier-Kulesa, Morgan H. Romine, Jason A. Morrison, Caleb M. Bailey, Danny R. Welch, Paul M. Kulesa

**Affiliations:** 1 Stowers Institute for Medical Research, Kansas City, MO 64110, USA; 2 Duke University, Margolis Center for Health Policy, Washington, DC 20004, USA; 3 Department of Biology, Brigham Young University-Idaho, Rexburg, ID 83460, USA; 4 Department of Cancer Biology, University of Kansas Medical Center, Kansas City, KS 66160, USA; 5 Department of Anatomy and Cell Biology, University of Kansas Medical Center, Kansas City, KS 66160, USA

**Keywords:** Nerve growth factor, Human, Melanoma, Metastasis, Chick embryonic microenvironment, Neural crest

## Abstract

Melanoma pathogenesis from normal neural crest-derived melanocytes is often fatal due to aggressive cell invasion throughout the body. The identification of signals that reprogram de-differentiated, metastatic melanoma cells to a less aggressive and stable phenotype would provide a novel strategy to limit disease progression. In this study, we identify and test the function of developmental signals within the chick embryonic neural crest microenvironment to reprogram and sustain the transition of human metastatic melanoma to a neural crest cell-like phenotype. Results reveal that co-culture of the highly aggressive and metastatic human melanoma cell line C8161 upregulate a marker of melanosome formation (Mart-1) in the presence of embryonic day 3.5 chick trunk dorsal root ganglia. We identify nerve growth factor (NGF) as the signal within this tissue driving Mart-1 re-expression and show that NGF receptors trkA and p75 cooperate to induce Mart-1 re-expression. Furthermore, Mart-1 expressing C8161 cells acquire a gene signature of poorly aggressive C81-61 cells. These data suggest that targeting NGF signaling may yield a novel strategy to reprogram metastatic melanoma toward a benign cell type.

## Abstract (precis)

**Summary:** We identify and test the function of nerve growth factor to reprogram human metastatic melanoma cells to a less aggressive phenotype. This article has an associated First Person interview with the first author of the paper as part of the supplementary information.

## INTRODUCTION

Multipotent neural crest cells migrate aggressively, but in a controlled manner, in discrete streams throughout the embryo to contribute to vertebrate organogenesis (Kulesa and Gammill, 2010). In the trunk, neural crest cells give rise to neurons and glia of the peripheral nervous system (PNS) and melanocytes in the skin (Le Douarin and Kalcheim, 1999). During assembly of the PNS, to exit the dorsal neural tube, the initial neural crest cells follow ventral pathways between the neural tube and somite and later through loosely connected somitic mesoderm. This migratory pattern results in the distribution of trunk neural crest cells into a ventral location to form the sympathetic ganglia (SG), and dorsal location to form the sensory dorsal root ganglia (DRG) (Kulesa et al., 2009). Later emerging trunk neural crest cells follow a dorsolateral migratory pathway and distribute throughout the surface ectoderm to differentiate into pigment cells that synthesize melanin. Thus, trunk neural crest cells exit from a common location but are directed to distinct peripheral locations and respond to local microenvironmental signals to build functional tissue architectures along the vertebrate posterior axis.

Signals within the trunk neural crest microenvironment that regulate the migration and differentiation of multipotent neural crest cells have been identified (Kulesa and Gammill, 2010). Experiments in chick showed that the CXCR4/CXCL12 signaling axis is critical to guiding trunk neural crest cells to the dorsal aorta where cells are sculpted into discrete primary sympathetic ganglia (Kasemeier-Kulesa et al., 2005, 2006, 2010; Saito et al., 2012). Studies within the chick have identified that TrkB and brain derived neurotrophic factor (BDNF) signals direct the sympathetic precursor cells to the secondary sympathetic ganglia site (Kasemeier-Kulesa et al., 2015). Later emerging trunk neural crest cells migrate along the ventral pathway but stop in a dorsal position to form the DRG, and within the DRG neural crest cells respond to several neurotrophic factors including nerve growth factor (NGF), neurotrophin-3 (NT-3) and BDNF. Thus, the embryonic trunk neural crest microenvironment is rich in the number of factors that strongly influence the guidance, differentiation and survival of cells to assemble the peripheral nervous system.

Heterotopic grafting experiments using the quail-chick chimera system and *in vivo* lineage tracing studies have concluded that the fate of trunk neural crest cells that form the PNS remains plastic until they receive differentiation signals at the end of, and possibly during, migration (Le Douarin et al., 1969; Le Douarin, 1980; Bronner-Fraser and Fraser, 1988, 1989; Raible and Eisen, 1994). The plasticity displayed by neural crest cells, most notably by neurons, glia, and melanocytes, makes the cells capable of responding to microenvironmental signals that play a role in differentiation and migration. For example, differentiated glia cells and melanocytes may reacquire the bipotent state of the original glial-melanocyte precursor. When single melanocytes from quail embryos are cultured in the presence of Endothelin-3 (Edn3), cells de-differentiate and activate glial-specific genes, giving rise to clonal progeny that contain glial cells and melanocytes (Dupin et al., 2000). Together, these data provide strong evidence for the plasticity of embryonic and adult neural crest cells, however it is not known whether this plasticity is a characteristic of a neural crest-derived cancer, such as melanoma.

We previously showed that the human melanoma cell line C8161 (highly aggressive and metastatic) transplanted into the chick embryonic neural crest microenvironment follow stereotypical neural crest cell migratory pathways, do not reform tumors, and re-express a melanocyte marker, Mart-1, in a small subset of invading cells (Kulesa et al., 2006; Hendrix et al., 2007). Western blot analysis revealed the presence of Mart-1 in the C81-61 (poorly aggressive) non-metastatic isogenic counterpart as well as the human melanocyte cell line HEMn, but not C8161 metastatic melanoma cells (Kulesa et al., 2006). We hypothesized that there is a signal(s) within the embryonic neural crest microenvironment capable of driving Mart-1 re-expression in de-differentiated metastatic melanoma cells. To test this, we combine co-culture assays, genomic profiling and *in vivo* imaging in chick. By generating a lentiviral Mart-1:GFP reporter, we possessed a dynamic means to evaluate metastatic melanoma reprogramming in the presence of developmentally staged chick tissues corresponding to the embryonic neural crest microenvironment. Through a series of co-culture experiments of human patient-derived C8161 metastatic melanoma cells with various chick head and trunk tissues and factors known to be present in these tissues, we sought to determine the precise microenvironmental location and source of the signal(s) capable of driving Mart-1 re-expression. We provide details of the dynamics and stability of Mart-1 re-expression and behaviors of C8161 Mart-1:GFP-positive metastatic melanoma cells. Our results identify the signal within the embryonic neural crest microenvironment capable of reprogramming the metastatic melanoma phenotype to a less aggressive glial-melanocyte cell type.

## RESULTS

### Generation of a lentiviral Mart-1:GFP reporter provided a dynamic readout of changes in Mart-1 expression

We previously showed that human C8161 metastatic melanoma cells transplanted into the chick embryo invade along host head and trunk neural crest pathways, do not reform tumors, and adopt a controlled invasion program similar to the host neural crest (Kulesa et al., 2006; Hendrix et al., 2007; Bailey et al., 2012). What was further intriguing was that a subset of transplanted C8161 metastatic melanoma cells upregulated Mart-1, a melanocyte differentiation marker (Serafino et al., 2004) involved in melanosome formation that is only present in the C81-61 non-metastatic isogenic counterpart (Kulesa et al., 2006). This provided us with a working hypothesis that signals within the embryonic chick neural crest microenvironment are capable of reprogramming a metastatic melanoma cell to a less aggressive neural crest cell-like phenotype.

**Fig. 1.**
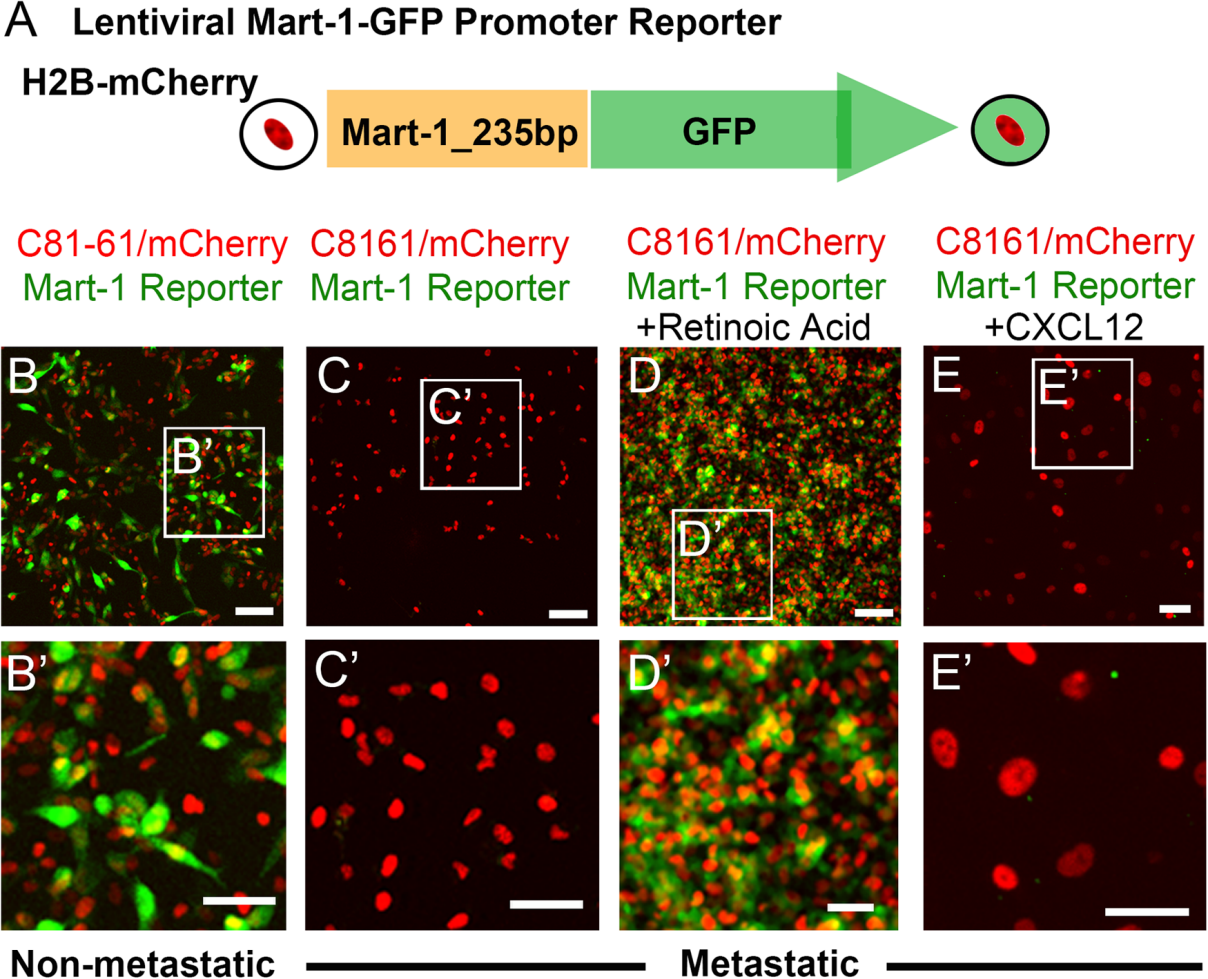
**Generation of a lentiviral Mart-1:GFP promoter reporter.** (A) A 235 bp sequence corresponding to the promoter sequence of human Mart-1 was cloned upstream of the GFP gene in the lentiviral plasmid. (B-E) Lentiviral infection of both (B-B′) C81-61 non-metastatic and (C-E) C8161 metastatic melanoma cells with Mart-1:GFP//h2B:mCherry. (B-B′) C81-61 cells express Mart-1:GFP (green) and h2b:mCherry (red). (C-C′) C8161 metastatic melanoma cells do not express Mart-1:GFP (green) but do express h2b:mCherry (red). (D-D′) C8161 cells in the presence of 10 μM of ATRA retinoic acid express Mart-1:GFP (green) (positive control). (E-E′) C8161 cells in the presence of the chemokine ligand CXCL12 do not express Mart-1:GFP (negative control). B-E repeated in triplicate. Scale bars (B-E): 100 μm; (B′-F′): 50 μm. GFP, green fluorescent protein.

To begin to test this, we sought to generate a fluorescent Mart-1:GFP reporter construct that, when introduced into cells, would provide a vital, dynamic readout of changes in Mart-1 expression. Using a Mart-1 reporter plasmid tested in melanoma cells (kind gift from Michihiro Konno, Nagoya University; Song et al., 2009), we generated a lentiviral Mart-1:GFP promoter reporter plasmid and stably infected both C8161 metastatic and C81-61 non-metastatic melanoma cell lines, co-labeled with nuclear localized H2B-mCherry (Fig. 1A). C81-61 non-metastatic melanoma cells that typically express Mart-1 showed robust fluorescence signal of Mart-1:GFP, indicating successful generation of our reporter construct (Fig. 1B-B′). Untreated C8161 metastatic melanoma cells showed no Mart-1:GFP fluorescence signal, confirming the lack of Mart-1 expression in these cells (Fig. 1C-C′). To analyze the function of the reporter construct in C8161 metastatic melanoma cells, we forced Mart-1 expression by exposure to retinoic acid (Serafino et al., 2004) and found Mart-1:GFP fluorescence signal throughout the cell line, as expected (Fig. 1D-D′). Exposure of C8161 metastatic melanoma cells to the chemokine ligand CXCL12 showed no Mart-1:GFP expression, as expected (Fig. 1E-E′).

### Co-culture of human C8161 metastatic melanoma cells with chick embryonic head and trunk tissues identified stage-dependent increases in Mart-1 re-expression

**Fig. 2.**
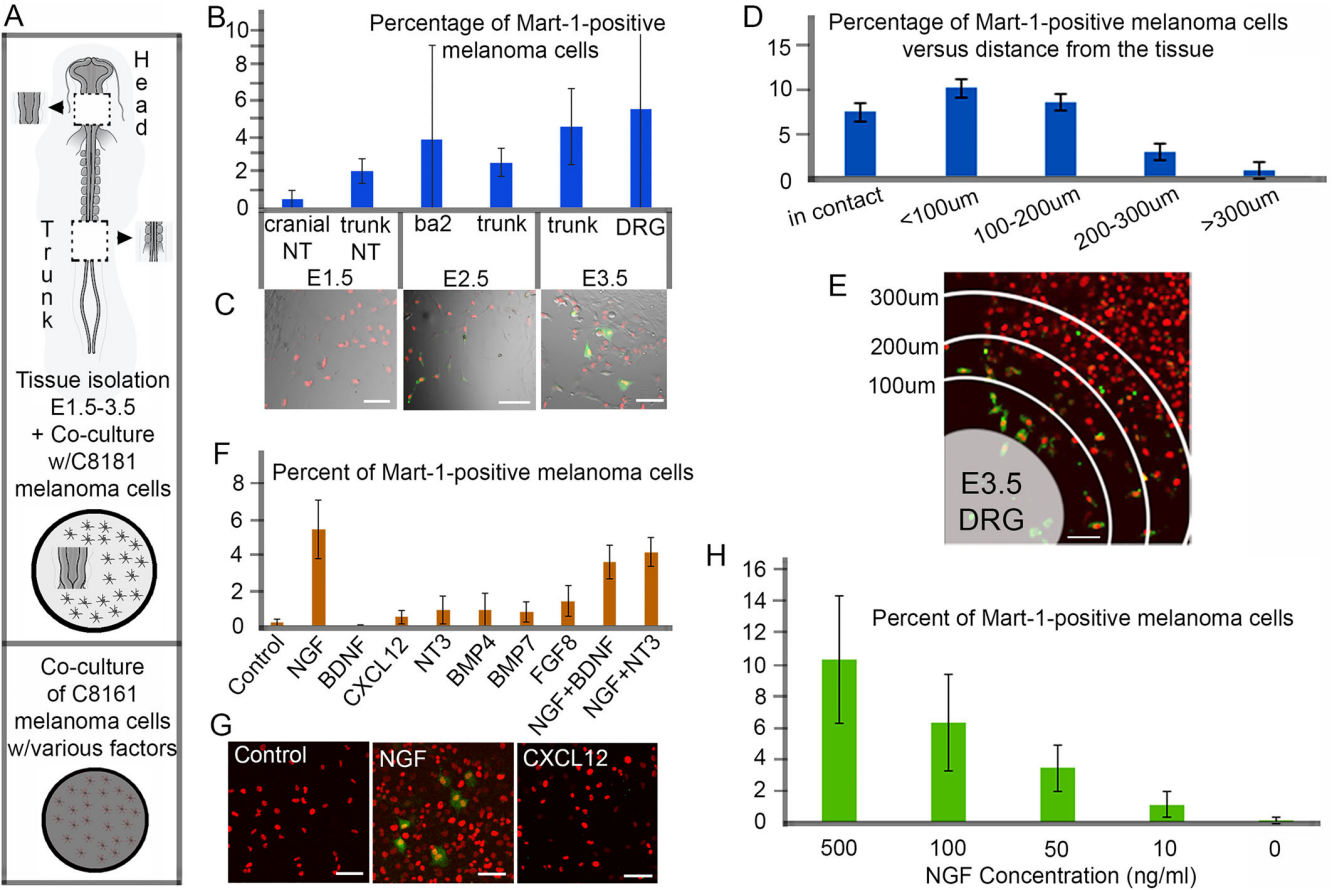
**Identification of E3.5 chick dorsal root ganglia tissue and nerve growth factor (NGF) drive Mart-1:GFP expression in human C8161 melanoma cells.** (A) Schematic of co-culture assays using plated human C8161 human melanoma cells with either varying ages and regions of chick embryonic tissues from the head or trunk (top) or soluble factors (bottom). (B) Percentage of Mart-1:GFP-positive C8161 melanoma cells after co-culture with different ages and regions of chick embryonic tissues (E1.5 cranial nt, 0.5±0.5%, s.d.; E1.5 trunk nt, 2.1±0.7%, s.d.; E2.5 ba2, 3.9±5.5%, s.d.; E2.5 trunk, 2.6±0.8%, s.d.; E3.5 trunk, 4.7±2.2%, s.d.; E3.5 DRG, 5.68±9.5%, s.d.). One section/piece of tissue was added per well of cells (8-well chamber slide format). Experiment was repeated in triplicate. (C) Typical images of C8161 melanoma cells (pre-labeled with H2B-mCherry, red) co-cultured with successive ages of trunk tissues, E1.5 (left), E2.5 (middle), and E3.5 (right) show an increase in Mart-1:GFP re-expression (green) with age. (D) Percentage of Mart-1:GFP-positive melanoma cells versus distance from the tissue (in contact with tissue 7.7±2.1 μm, s.d.; ≤100 μm, 10.5±3.3 μm, s.d.; 100-200 μm, 8.8±3.4 μm, s.d.; 200-300 μm, 3.1±2.7 μm, s.d. and >300 μm, 0.8±0.7 μm, s.d.). Experiment was repeated in triplicate. (E) A typical image of C8161 melanoma cells (pre-labeled with H2B-mCherry, red) co-cultured with E3.5 DRG (bottom left in E) show higher Mart-1:GFP re-expression closer to transplanted DRG tissue (100 μm increments from the edge of the tissue are labeled). (F) Percentage of Mart-1:GFP-positive C8161 melanoma cells in the presence of individual soluble factors (Control, no factor 0.2±0.2%, s.d.; NGF, 5.46±1.65%, s.d., *P*<0.01; BDNF, 0.05±0.04%, s.d., *P*=0.2; CXCL12, 0.5±0.4%, s.d., *P*=0.3; NT3, 0.9±0.8%, s.d., *P*=0.3; BMP4, 0.93±0.9%, s.d., *P*=0.1; BMP7, 0.8±0.58%, s.d., *P*=0.1; FGF8, 1.4±0.86%, s.d., *P*=0.7; NGF+BDNF, 3.6±0.9%, s.d.; NGF+NT3, 4.17±0.8%, s.d.) typically found in E3.5 trunk tissue. Experiment was repeated in triplicate. (G) A typical image of C8161 melanoma cells (pre-labeled with H2B-mCherry, red) in the presence of no factor (left), NGF (middle) and Cxcl12 (right) and Mart-1:GFP re-expression (green). (H) Percentage of Mart-1:GFP-positive C8161 melanoma cells exposed to different concentrations of NGF (500 ng/ml, 10.3±4.03%, s.d., *P*<0.01; 100 ng/ml, 6.3±3.1%, s.d., *P*=0.2; 50 ng/ml, 3.4±1.5%, s.d., *P*=0.02; 10 ng/ml, 1.1±0.8%, s.d., *P*=0.1; 0 ng/ml, 0.1±0.2%, s.d.). Experiment was repeated in triplicate. Scale bars: 50 μm. All calulations performed on fixed samples. Statistical analysis was performed using Student's *t*-test.

To determine the time and location of the chick embryonic neural crest microenvironment signal(s) capable of driving Mart-1 re-expression in C8161 metastatic melanoma cells, we took advantage of co-culture assays and the fluorescent readout of Mart-1 expression. C8161 cells co-labeled with Mart-1:GFP//H2B-mCherry and cultured in chamber slides were initially exposed to either head or trunk tissues isolated at increasing embryonic stages (HHSt10, 15, 17, and 21; Hamburger and Hamilton, 1951) for 72 h, and Mart-1:GFP-positive melanoma cells were counted (Fig. 2A). We found that the percentage of Mart-1:GFP-positive melanoma cells increased with increasing age of co-cultured tissue (Fig. 2B,C). Specifically, we discovered that co-culture with HHSt21 [embryonic day (E)3.5, DRG] trunk tissue showed that 5% of C8161 metastatic melanoma cells re-expressed Mart-1 (Fig. 2B). We noticed large error bars, specifically in the presence of E2.5 BA2, E3.5 trunk and E3.5 DRG tissues. Further analysis showed that proximity of the cells to the co-cultured E3.5 DRG tissue affected the percent of Mart-1:GFP-positive melanoma cells (Fig. 2D,E). C8161 cells in contact with the tissue and within 200 μm showed Mart-1-positive re-expression at ∼9% (in contact 7.7±2.1% s.d., within 100 μm 10.5±3.3% s.d., *P*=0.2 and 100-200 μm 8.8±0.7% s.d., *P*=0.4 were not statistically different), and this decreased at 200-300 μm away to 3.1±2.7% (s.d.) (*P*=0.08, versus in contact), and greater than 300 μm to 0.8±0.7% (s.d.) (*P*<0.01, versus in contact; Fig. 2D,E).

### NGF produced a significant increase in Mart-1 re-expression in C8161 metastatic melanoma cells when tested in co-culture with six other embryonic neural crest microenvironmental factors

At HHSt21, trunk neural crest cells have migrated to form the ventrally positioned SG, the dorsally positioned DRG, and populated the dorsolateral pathway to give rise to melanocytes in the skin. Also at this developmental stage and locations of neurogenesis, the embryonic neural crest microenvironment is rich in trophic factors and differentiation signals. This includes neurotrophins responsible for DRG formation and differentiation (BDNF, NT3, NGF) and SG-derived signals (CXCL12, BMP4, BMP7, FGF8). When we exposed C8161 metastatic melanoma cells to each of these factors separately for 72 h, we found that NGF produced a significant increase in Mart-1 re-expression (Fig. 2F,G). Repeating the co-culture experiments with two distinct combinations (NGF+BDNF, NGF+NT3) of the seven factors did not increase the re-expression over what was measured with NGF (Fig. 2F). Furthermore, when we varied the concentration of NGF in co-culture assays, we discovered increasing concentrations of NGF led to a higher percentage of Mart-1:GFP-positive melanoma cells; 500 ng/ml versus 50 ng/ml of NGF doubled the percentage of Mart-1-positive melanoma cells from 3.4±1.5% (s.d.) to 10.3±4.03% (s.d.) (*P*<0.05; Fig. 2H).

### NGF did not alter the proliferation or attract C8161 metastatic melanoma cells

**Fig. 3.**
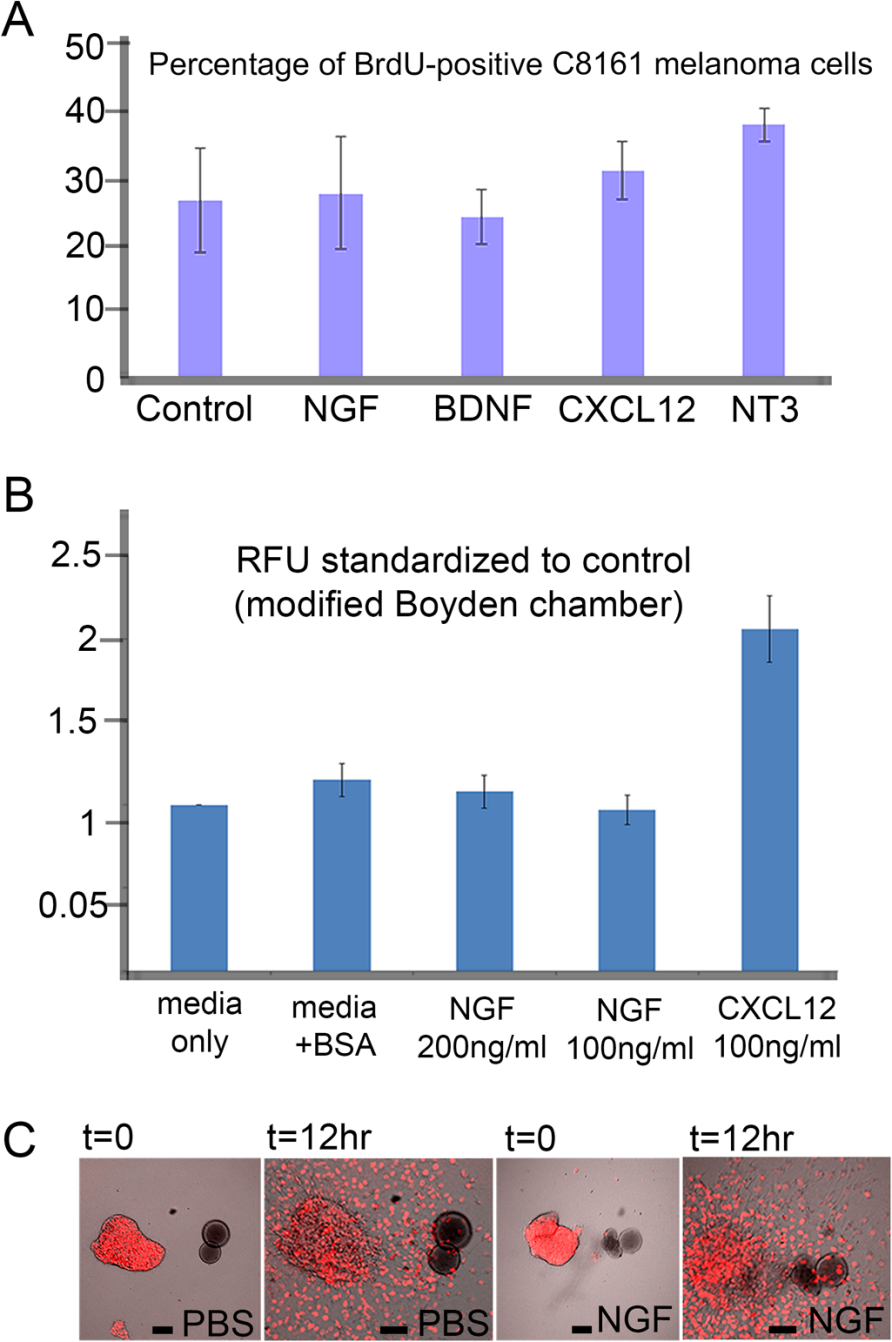
**NGF does not affect proliferation or migration of C8161 melanoma cells.** (A) Percentage of BrdU-positive C8161 melanoma cells under different co-culture conditions with various soluble factors (Control, no factor 26.9±7.86 cells, s.d.; NGF, 28±8.6 cells, s.d., *P*=0.5; BDNF, 24.4±4.17 cells, s.d., *P*=0.6; CXCL12, 31.5±4.42 cells, s.d., *P*=0.4; NT3, 38.4±2.5, s.d., *P*=0.07). Experiment was repeated in triplicate. (B) C8161 melanoma cells seeded into a modified Boyden chamber show no preference for BSA control (1.15±0.1 s.d., RFU) or NGF (200 mg/ml with 1.08±0.1 s.d., RFU, *P*=0.4 and 100 ng/ml with 0.97±0.09 s.d., RFU, *P*=0.2), but migrate in response to a known chemokine, CXCL12 (2.06±0.2 s.d., RFU, *P*<0.01). Experiment was repeated in duplicate. (C) PBS- or NGF-soaked beads co-cultured *in vitro* with C8161 melanoma cells (labeled with H2B-mCherry, red) and observed at t=0 and 12 h show no directed cell migration towards either the PBS- or NGF-soaked beads, *n*=4 bead cultures per condition, repeated in triplicate. Scale bars: 100 μm (t=0) and 50 μm (t=12 h). All calculations performed on living samples. Statistically analysis was performed using Student's *t*-test.

To test whether NGF altered the proliferation of C8161 metastatic melanoma cells, we measured changes in proliferation in co-culture experiments with NGF, BDNF, CXCL12 and NT3 by BrdU incorporation (Fig. 3A; 30-min pulse). We found no significant difference between control (26.9±7.9 cells, s.d.) and NGF-treated cultures (28±8.6 cells, s.d., *P*=0.5; Fig. 3A). However, a slight increase was seen in NT3 treated co-cultures, which was not statistically significant (38.4±2.5 cells, s.d., *P*=0.07; Fig. 3A). We then tested the ability of NGF to attract C8161 metastatic melanoma cells by challenging plated spheres of cells with either a PBS or NGF-soaked bead (Fig. 3C). There was no significant preference for either the PBS or NGF-soaked bead and cells spread in a uniform radial pattern from their plated sphere site (Fig. 3C). Furthermore, there was no difference between the percentage of cells in PBS [1.15±0.1,s.d., relative fluoresce units (RFU)] versus NGF (1.08±0.1, s.d., RFU, 200 mg/ml, *P*=0.2) wells of a modified Boyden chamber to assess NGF to attract C8161 cells (Fig. 3B). We did, however, find directed migration of C8161 melanoma cells towards CXCL12-soaked beads in culture and a high percentage of cells surrounding the bead, confirming the invasive ability of these cells (data not shown). In addition, 100 ng/ml of CXCL12 doubled the percentage of cells migrating into the lower well of a modified Boyden chamber (2.06±0.2, s.d., RFU, *P*<0.01; Fig. 3B). Additionally, no changes in morphology or behavior of the cells was noted.

### C8161 metastatic melanoma cells exposed to prolonged NGF treatment showed stable Mart-1:GFP re-expression

**Fig. 4.**
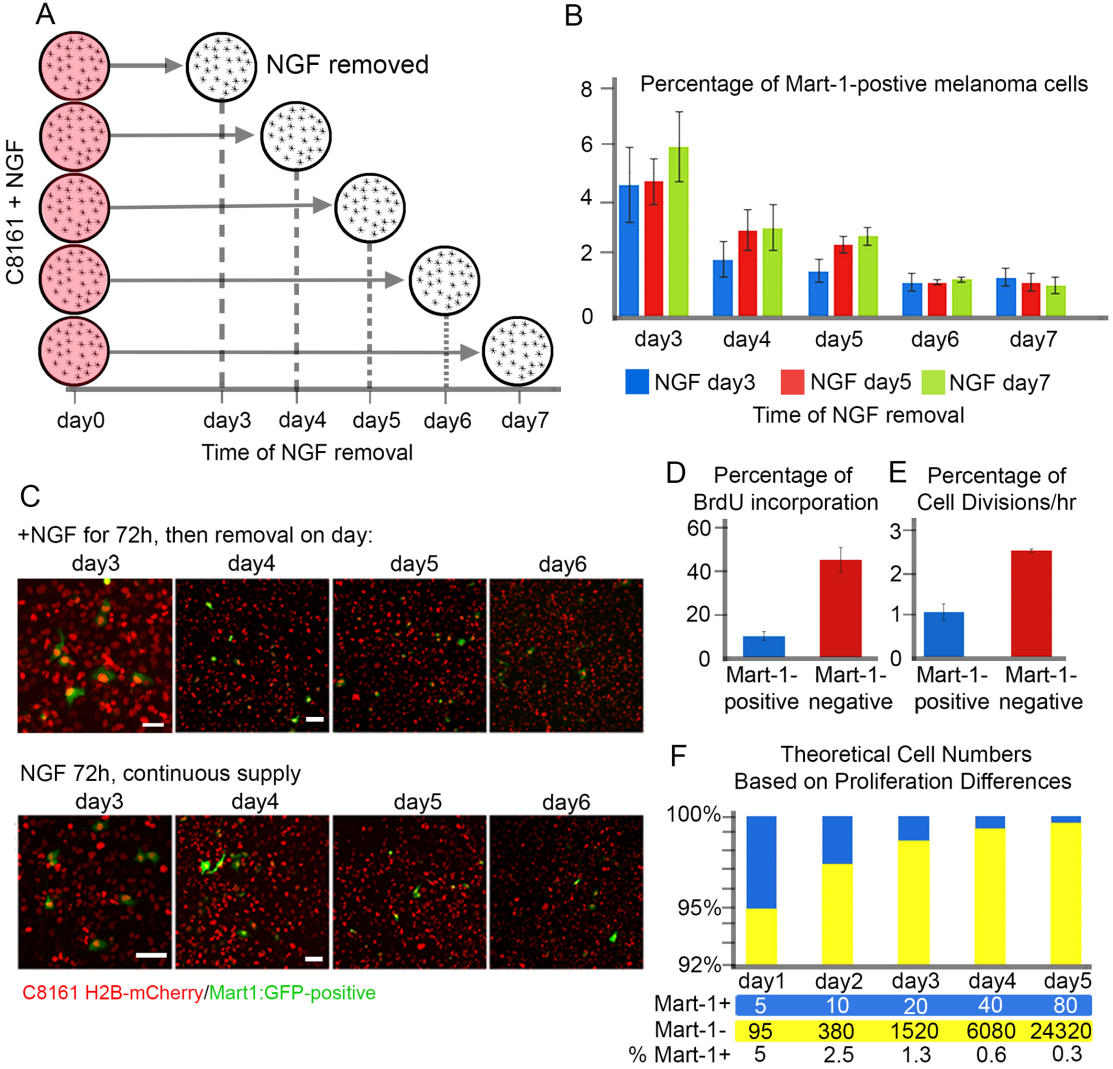
**NGF induces stable Mart-1:GFP-positive C8161 melanoma cell population.** (A) Schematic of the NGF time course experiment. At day 0, all plates of C8161 cells received NGF (circles shaded red) and cultured for at least 3 days before removal of NGF (white circle). On successive days (*x*-axis) the NGF was removed. (B) Percentage of Mart-1:GFP melanoma cells in the presence of NGF for at least 3 days and then removal on day3 (blue), day 5 (red), day 7 (green). Within each day, no data set was significantly different from the others (Day 3: NGF 3 days=4.6±1.3%, s.d.; NGF 5 days, 4.7±0.8%, s.d.; NGF 7 days, 5.9±1.2%, s.d.; Day 4: NGF 3 days, 1.8±0.6%, s.d.; NGF 5 days, 3±0.7%, s.d.; NGF 7 days, 3.1±0.8%, s.d.; Day 5: NGF 3 days, 1.6±0.4%, s.d.; NGF 5 days, 2.5±0.3%, s.d.; NGF 7 days, 2.8±0.3%, s.d.; Day 6: NGF 3 days, 1.2±0.3%, s.d.; NGF 5 days, 1.2±0.1%, s.d.; NGF 7 days, 1.3±0.1% s.d.; Day 7: NGF 3 days, 1.37±0.3%, s.d.; NGF 5 days, 1.2±0.3%, s.d.; NGF 7 days, 1.1±0.3%, s.d.; at least 1000 cells counted per condition; each condition repeated in duplicate). (C) (Top Panel) C8161 melanoma cells after co-culture with NGF for 3 days (72 h) show ∼5% Mart-1:GFP-positive melanoma cells, then removal on subsequent days. A decrease in Mart-1:GFP-positive melanoma cells is seen on subsequent days (4-6). (Bottom Panel) C8161 melanoma cells maintained in culture with constant supply of NGF show same ∼5% Mart-1:GFP-positive melanoma cells at day 3 and similar decrease in Mart-1:GFP-positive cells at days 4-6. (D) Proliferation rate of C8161 melanoma cells changes after Mart-1:GFP expression. Percentage of BrdU incorporation in C8161 cells after 30 min BrdU pulse: Mart-1:GFP-positive melanoma cells, 10±2.3% (s.d.) and Mart-1:GFP-negative melanoma cells, 45.5±3.4% (s.d.), *P*<0.01 (left graph; *n*>500 cells counted per condition, experiment run in duplicate). (E) Time-lapse culture analysis shows Mart-1 positive cells proliferate less (1.1±0.4 cell divisions per hour) than Mart-1-negative cells [2.56±0.3 (s.d.) cell divisions per hour], *P*<0.01. *n*=4 time lapses per condition. (F) Theoretical calculation of cell populations based on differences in proliferation dynamics. After NGF exposure, ∼5% of C8161 melanoma cells re-express Mart-1:GFP and the cell proliferation rate decreases. A doubling rate of 2 (cell cycles in 24 h) is applied to the Mart-1:GFP-positive cell population starting with 5 out of 100 cells and a rate of 4 (cell cycles in 24 h) is applied to the Mart-1-negative population starting at 95 out of 100 cells. Using a 24-h doubling time, the projected changes in cell population distribution are seen by a decrease in blue cell (Mart-1:GFP-positive) and increase in yellow cell (Mart-1:GFP-negative) populations. Scale bars: 50 μm. All calculations performed on living samples. Statistically analysis was performed using Student's *t*-test.

To determine whether Mart-1 re-expression in C8161 metastatic melanoma cells could be extended beyond our initial observations of 72 h, we tested whether removal of NGF would affect the number of Mart-1:GFP-positive cells (Fig. 4A). To address this, we set up a time course experiment in which C8161 melanoma cells were exposed to NGF (200 ng/ml) for either 3 days (blue bar), 5 days (red bar) or 7 days (green bar), and counted Mart-1:GFP-positive cells every 24 h (Fig. 4A,B). After 3 days, all cultures showed approximately 5% of C8161 metastatic melanoma cells re-expressed Mart-1 as expected (Fig. 4B). NGF was removed from one set of cultures (blue bar), and the other cultures were supplemented with fresh media and NGF (Fig. 4B). By day 4, cultures that had NGF removed 24 h previously (blue bar) showed a dramatic drop in Mart-1-positive cells (Fig. 4B; 2±0.6%, s.d., *P*=0.03 compared to blue bar on day 3).

Intriguingly, cultures that continued to be exposed to NGF also decreased the percentage of Mart-1-positive cells, but to a lesser extent (Fig. 4B). By day 5, the trend was the same with cells that had NGF removed 48 h previously, declining the percentage of Mart-1:GFP-positive cells (Fig. 4B) and cells with the continuous supply of NGF decreased to a lesser extent (Fig. 4B). After observation, NGF was removed from the 5 day cultures (red bar) and media and NGF replaced in the 7 day NGF cultures (Fig. 4B). On day 6 there was no difference in the percentage of Mart-1:GFP-positive cells in any of the cultures (Fig. 4B). That is, cells exposed to NGF for 3 days, 5 days or still exposed, decreased Mart-1 expression to roughly 1.5% (Fig. 4B). By day 7, there was no change in the percentage of Mart-1-positive cells from day 6, where all co-culture conditions leveled off to 1.5% Mart-1 re-expression (Fig. 4B; *P*=0.5 for blue and red bars at day 7 and *P*=0.3 for blue and green bars at day 7). Typical images show the changes in Mart-1:GFP expression (Fig. 4C).

### NGF exposure significantly decreased the proliferation of Mart-1:GFP-positive cells to a rate similar to C81-61 non-metastatic melanoma cells

There is a significant difference in proliferation rate between C8161 metastatic versus C81-61 non-metastatic melanoma cells. C8161 metastatic melanoma cells proliferate twice as fast as C81-61 non-metastatic cells (data not shown). To determine whether cell proliferation changed after Mart-1 re-expression, we assessed BrdU incorporation in C8161 metastatic melanoma cells (Fig. 4D). That is, after co-culturing C8161 metastatic melanoma cells with NGF (72 h) followed by a BrdU pulse for 2 h, we found a dramatic decrease in BrdU-positive Mart-1:GFP-positive cells compared to Mart-1:GFP-negative cells (Fig. 4D). Mart-1:GFP-positive C8161 metastatic melanoma cells showed a 10±2.3% (s.d.) BrdU incorporation, whereas Mart-1:GFP-negative cells produced a 45±3.4% (s.d., *P*<0.01) BrdU incorporation (Fig. 4D). To further confirm these results we tracked C8161 metastatic melanoma cells over time and compared the number of cell divisions (Fig. 4E). We found that Mart-1:GFP-negative C8161 melanoma cells produced 2.56±0.3 (s.d.) cell division per hour, 2.5 more divisions than Mart-1:GFP-positive cells (1.1±0.4, s.d., *P*<0.01; Fig. 4E). Taking these proliferation changes into consideration, a simple mathematical model showed that given an initial subpopulation of 5% Mart-1-positive cells cycling at a slower rate, the Mart-1-negative cells would expand faster and produce similar ratios of Mart-1-positive and Mart-1-negative cells. This indicated that the initial Mart-1:GFP-positive melanoma cell subpopulation is stable, but the overall percentage of Mart-1:GFP-positive cells decreased due to the higher proliferation rate of Mart-1:GFP-negative cells within the same culture (Fig. 4F).

### Mart-1:GFP-positive cell stability increased with multiple rounds of sorting

With the observation of significant proliferation differences between Mart-1:GFP-positive and Mart-1:GFP-negative C8161 metastatic melanoma cells, we used flow cytometry to determine whether sorting of Mart-1:GFP-positive cells after exposure to NGF (72 h) would maintain long-term Mart-1 expression (Fig. S1). After 72 h of co-culture with NGF (200 ng/ml), we determined a 5% re-expression of Mart-1 based on GFP signal. These cells were fluorescence-activated cell sorted (FACS) for continued culture. At 3 h post-sorting, we confirmed that ∼95% of cells were Mart-1:GFP-positive (Fig. S1). After culturing the FACS Mart-1:GFP-positive cells for 3 days, we determined that only 8% of the cells were Mart-1:GFP-positive (Fig. S1). Twenty-four hours later (4 days post-sorting), the cells decreased to 1.2% Mart-1-GFP-positive (Fig. S1).

By day 5, there was minimal change with 1.4% Mart-1:GFP-positive cells; this population was then sorted (a second time) and GFP-positive cells were continued in culture. At 3 h post sort we confirmed ∼95% were GFP positive. As before, 5 days after the second round of sorting, only 8% of cells were Mart-1:GFP-positive (Fig. S1). However, 9 days later (a total of 14 days after the second sort), this population was maintained at roughly 8% (Fig. S1). After 4 more days (18 days post second sort) this population decreased to ∼5% and these cells were then sorted a third time. At 24 h after the third round of sorting, ∼90% were Mart-1:GFP-positive and similar results observed at 3 days post third round of sorting (Fig. S1). Interestingly, 6 days after the third round of sorting we began to see a decrease in the Mart-1-positive population with ∼70% Mart-1:GFP-positive and a small decrease to ∼58% at 7 days post third round of sorting. Thus, multiple rounds of Mart-1 GFP-positive sorting identified a trend to a stable percent of Mart-1:GFP-positive cells to ∼8% over two weeks after the initial sort.

### NGF receptors trkA and p75 are expressed at significantly different levels in C8161 metastatic versus C81-61 non-metastatic melanoma cells

**Fig. 5.**
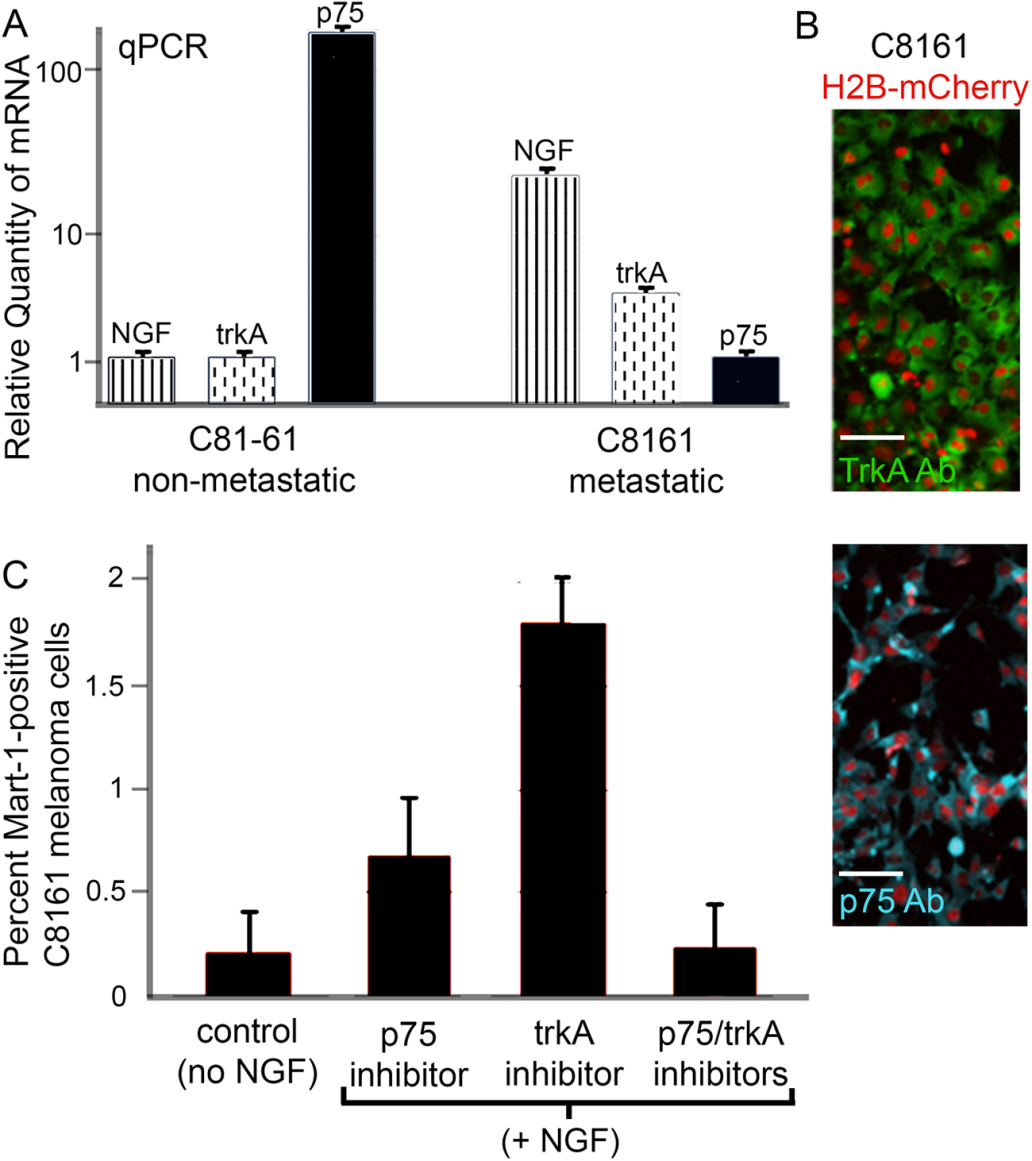
**NGF receptor expression on C8161 melanoma cells.** (A) qPCR analysis of relative quantity of mRNA differences of NGF (verticle striped bars), trkA (verticle dashed bars) and p75 (solid black bars) on C81-61 and C8161 melanoma cells. C81-61: NGF=1.00±0.06 s.d., trkA=1.00±.05 s.d., p75=170±8.7 s.d.; C8161: NGF=23.3±3.1 s.d., trkA=3.99±0.25 s.d., p75=1.00±0.15 s.d.; calibrated normalized relative quantities. (B) Protein expression of trkA (green, top panel) and p75 (blue, bottom panel) on H2B:mCherry labeled C8161 cells (red). (C) Percent of Mart-1:GFP-positive C8161 cells in control (no NGF) or in the presence of NGF and either p75 or trkA inhibitors individually or in combination (Control, 0.17±0.15%, s.d.; p75 inhibitor, 0.7±0.3%, s.d, *P*=0.05; trkA inhibitor, 1.8±0.2%, s.d., *P*<0.01; p75/trkA inhibitors, 0.2± 0.2%, s.d., *P*=0.8). At least 2000 cells counted per condition. Scale bars: 50 μm. Statistical analysis was performed using Student's *t*-test.

To determine whether C8161 metastatic and C81-61 non-metastatic melanoma cells express the NGF receptors, trkA (high affinity) and p75 (low affinity), we used a qPCR approach (Fig. 5A). We found that both trkA and p75 are expressed by both subpopulations of cells but at very different levels (Fig. 5A). trkA was expressed higher in C8161 metastatic versus C81-61 non-metastatic melanoma cells (Fig. 5A). The converse was true for p75 being higher in C81-61 non-metastatic compared to C8161 metastatic melanoma cells (Fig. 5A). Interestingly, both cell lines expressed NGF, with higher expression in C8161 metastatic versus C81-61 non-metastatic melanoma cells (Fig. 5A). After determining RNA expression of the receptors, we used immunohistochemistry to determine the protein expression of trkA and p75 and determined robust expression of both trkA and p75 on C8161 metastatic melanoma cells (Fig. 5B).

### Inhibition of NGF receptors decreased Mart-1 re-expression in C8161 metastatic melanoma cells

After determining that C8161 metastatic melanoma cells express both NGF receptors, we used inhibitors to block each receptor individually, and in combination in co-culture experiments in the presence of NGF, and assessed the extent of Mart-1:GFP re-expression (Fig. 5C). As expected, we observed a decrease in Mart-1:GFP re-expression when either receptor was blocked, but a more dramatic decrease when p75 was blocked (0.7±0.3%, s.d., Mart-1+ cells, *P*=0.05 versus no NGF control) versus trkA (1.8±0.2%, s.d., *P*<0.01; Fig. 5C). When both receptors were blocked simultaneously, we observed very few Mart-1:GFP-positive C8161 metastatic melanoma cells (0.2±0.2%, s.d., *P*=0.8; Fig. 5C), similar to control conditions without NGF (0.17±0.15%, s.d.; Fig. 5C). These results indicate the importance for both receptors in NGF-induced Mart-1:GFP re-expression.

### Mart-1:GFP-positive C8161 metastatic melanoma cells exposed to NGF showed high p75 and low TrkA expression, characteristic of the C81-61 non-metastatic counterpart

**Fig. 6.**
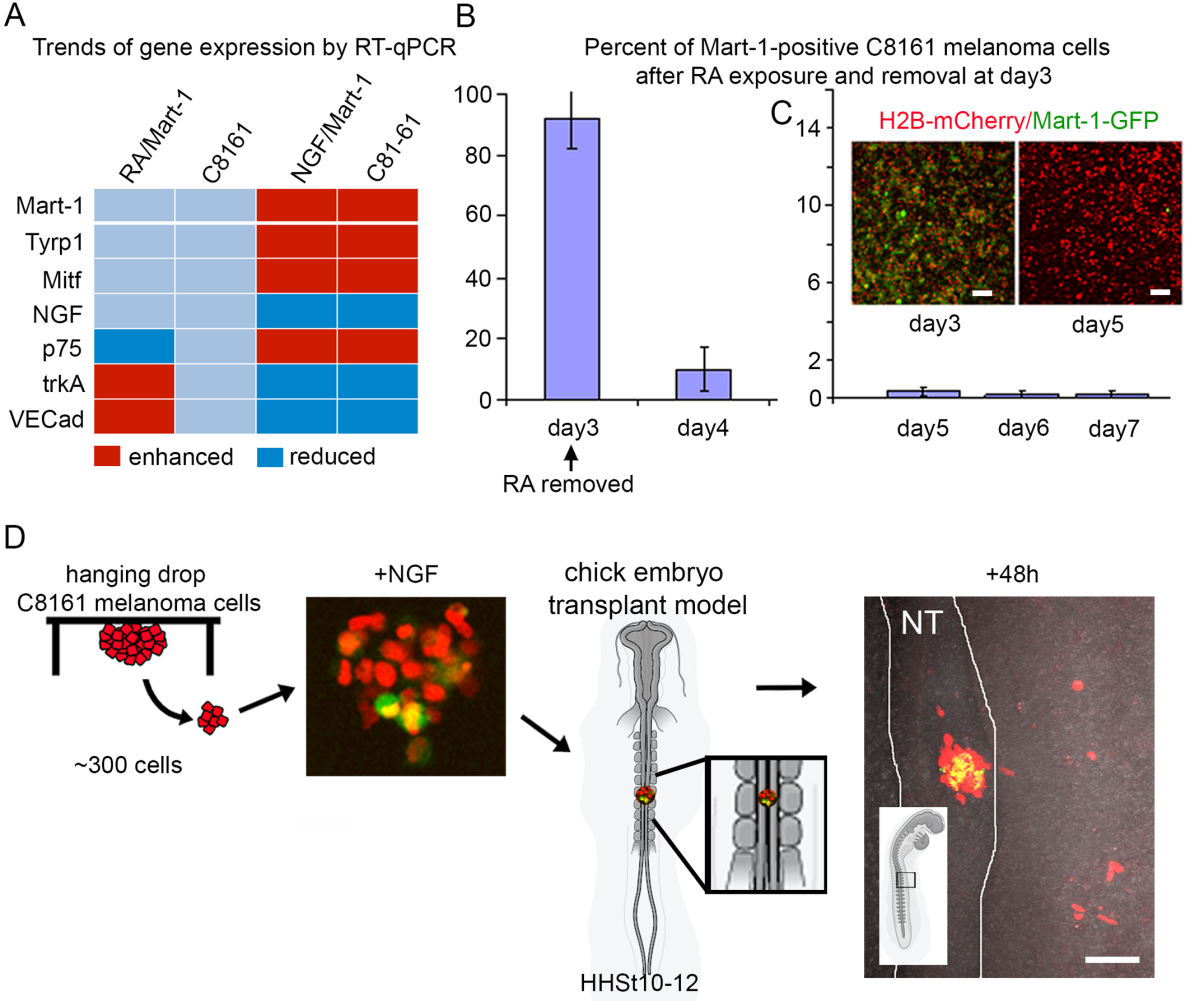
**Gene profiling and phenotypic analysis of NGF versus RA-induced Mart-1:GFP-positive C8161 melanoma cells.** (A) Gene expression comparison of Mart-1:GFP-positive C8161 metastatic melanoma cells after RA and NGF exposure compared to wild-type C8161 metastatic melanoma cells (light blue) and C81-61 non-metastatic melanoma cells. Increased (shown in red) and decreased (shown in dark blue) expression above/below wild-type levels (shown in light blue) are based on qPCR values. (B) C8161 metastatic melanoma cells in the presence of RA for 3 days show nearly all cells express Mart-1:GFP (93±8.4%, s.d.), and only 8.1±6.7% (s.d.; *P*<0.01) 24 h after removal of RA (day 4). Experiment was repeated in duplicate. (C) Further decline in Mart-1:GFP expression continues after RA removal, analyzed on days 5-7. (C, inset) C8161-H2B:mCherry/Mart-1:GFP-positive melanoma cells after 3 days of RA exposure (left panel), and 48 h after RA removal (right panel). Day 5=0.34±0.26 s.d., day 6=0.21±0.19% s.d., day 7=0.2±0.2 s.d. (D) Schematic of hanging drop culture of C8161 melanoma cells pre-labeled with H2B-mCherry and then exposed to NGF to induce ∼5% Mart-1:GFP re-expression and transplanted into the chick neural tube at HHSt12. After 48 h of egg re-incubation, C8161 melanoma cell positions were analyzed and included cell invasion into the periphery away from the outline of the neural tube transplant site or remained at the transplant site. Mart-1:GFP-positive cells appeared to be confined to the initial transplant site within the neural tube. *n*=5 embryo transplants. NT, neural tube. Scale bars: 50 μm. Calculations done on living samples (B,C). Statistical analysis was performed using Student's *t*-test.

After co-culturing C8161 metastatic melanoma cells with NGF for 72 h, we isolated Mart-1:GFP-positive re-expressing C8161 cells by FACS and compared gene expression profiles to wild-type C8161 and C81-61 cells using qPCR (using C8161 as baseline expression, light blue bar; Fig. 6A). The most striking difference of Mart-1:GFP-positive C8161 metastatic melanoma cells as compared to wild-type C8161 cells was expression of the NGF receptor p75 (Fig. 6A). Mart-1:GFP-positive C8161 metastatic melanoma cells had increased levels of p75, similar to C81-61 cells (Fig. 6A); trkA was also decreased, simliar to C81-161 cell expression (Fig. 6A). The high p75 and low trkA expression profile was indicative of the expression pattern in C81-61 non-metastatic melanoma cells (high p75 and low trkA; Figs 6A and 5A).

### NGF induced gene expression changes distinct from retinoic acid in Mart-1:GFP-positive C8161 metastatic melanoma cells

Are the gene expression changes induced from NGF versus RA induced Mart-1:GFP re-expression in C8161 metastatic melanoma cells similar? To determine this, we compared expression changes in NGF receptors after NGF or RA exposure. RA-induced Mart-1:GFP-positive cells decreased their p75 expression (Fig. 6A), whereas above, we found NGF caused an increase in p75 expression. Furthermore, RA-induced Mart-1:GFP-positive cells increased their trkA expression (Fig. 6A), whereas above, we found NGF decreased trkA expression. These results indicate that both NGF and RA can turn on Mart-1 expression, but downstream gene expression changes are different.

Interestingly, trends in gene expression changes showed that NGF-induced Mart-1:GFP-positive cells were similar to C81-61 non-metastatic melanoma cells, and RA-induced Mart-1:GFP-positive cells showed very little change from C8161 cells. Other genes including melanocyte markers Mart-1, Tyrp1 and Mitf (all of which are down-regulated in metastatic versus non-metastatic C8161 melanoma cells), showed that NGF-induced Mart-1:GFP-positive cells aligned with C81-61 expression profiles and RA-induced Mart-1-positive cells aligned with wild-type C8161 cell expression (Fig. 6A). Furthermore, C8161 metastatic melanoma cells exposed to RA for 3 days show a dramatic decrease in the percentage of Mart-1:GFP-positive cells to negligible levels after RA removal (Fig. 6B,C). Taken together, NGF-induced Mart-1:GFP-positive cells showed gene expression changes similar to C81-61 non-metastatic melanoma cells in contrast to RA-induced Mart-1:GFP-positive melanoma cells that showed no overall gene expression changes from wild-type C8161 melanoma cells, and only a transient spike in Mart-1:GFP expression, indicating RA turns on the Mart-1:GFP reporter but is insufficient to drive Mart-1 protein expression.

### Mart-1:GFP-positive C8161 metastatic melanoma cells failed to invade after transplantation into the chick embryonic neural crest microenvironment

C8161 metastatic melanoma cells aggressively invade the chick embryonic neural crest microenvironment in both the head and trunk (Kulesa et al., 2006; Bailey et al., 2012; Bailey and Kulesa, 2014). To determine whether re-expression of Mart-1 in C8161 metastatic melanoma cells affected their invasiveness, we transplanted these cells into the embryonic chick trunk neural crest microenvironment (Fig. 6D). For transplantation purposes, we added NGF to hanging drop cultures of C8161 metastatic melanoma cells (Fig. 6D). Surprisingly, after 48 h in hanging drops, we noted 18.1±2.52% of C8161 cells expressing GFP, an over threefold increase of Mart-1:GFP cells than observed in plated co-culture experiments (Fig. 6D). We then transplanted subgroups of C8161 metastatic melanoma cells cut from the hanging drops into the embryonic chick neural crest microenvironment (dorsal neural tube of HH St10 embryos) and re-incubated eggs for 72 h. Interestingly, we found that the majority of Mart-1:GFP-positive C8161 metastatic melanoma cells did not exit the dorsal neural tube and invade embryonic chick neural crest microenvironment (Fig. 6D). In contrast, Mart-1:GFP-negative C8161 metastatic melanoma cells from the same transplant invaded the host chick embryo along stereotypical neural crest cell migratory pathways (Fig. 6D).

## DISCUSSION

We used the chick embryo transplant model to study the reprogramming of human metastatic melanoma cells towards a benign cell type. We had previously reported that human patient-derived C8161 metastatic melanoma cells upregulated a marker of melanin synthesis, Mart-1, after exposure to unknown signals in the embryonic neural crest microenvironment (Kulesa et al., 2006). The goal of this study was to identify and examine the function of the microenvironmental signal(s) underlying the reprogramming process. To enable the dynamic readout of one of the changes in metastatic melanoma cell state, we generated a lentiviral Mart-1:GFP reporter and methodically determined the age, tissue type and ultimately the factor that induced re-expression of Mart-1. We learned that the neurotrophin NGF induced Mart-1 re-expression and changes in cell behavior and gene expression of human C8161 metastatic melanoma cells. We confirmed Mart-1:GFP re-expression in C8161 cells after NGF exposure using Mart-1 antibody staining (Fig. S1). Furthermore, we discovered that Mart-1 reprogrammed cells up- and down-regulated genes associated with non-metastic melanoma cells, and failed to invade the tissue when transplanted into the chick embryonic neural crest microenvironment.

Our finding that NGF induced re-expression of key regulators in melanin production in amelanotic C8161 metastatic melanoma cells was suggestive of reprogramming towards a benign multipotent neural crest cell type. Melanocytes, neurons and glia of the autonomic nervous system originate from multipotent neural crest cells during development (Le Douarin and Kalcheim, 1999). Glial-melanocyte precursor cells respond to Edn3 signals that increase cell proliferation and direct cell differentiation into melanocytes (Le Douarin et al., 2004). Furthermore, the glial-melanocyte cell state may be reacquired by adult differentiated pigment cells cultured in the presence of Edn3 (Dupin et al., 2000). Neurotrophins that regulate proliferation, survival and differentiation of neural crest-derived neuronal precursors also have roles during melanocyte development. Although neurotrophins do not stimulate melanocyte proliferation, they do stimulate the synthesis of tyrosinase and tyrosine-related protein-1, and NGF specifically increases melanin production in melanocytes (Marconi et al., 2006). Future studies may determine whether reprogrammed C8161 metastatic melanoma cells produce pigment and adopt a melanocyte-like function. Thus, together, our results support the plasticity of embryonic and adult melanocytes, and add that adult melanoma cells possess the plasticity to return to the neuron-glial-melanocyte precursor cell type.

The control and balance of the p75 receptor through NGF expression may be a potential target for reprogramming de-differentiated and aggressive neural crest-derived cancers to a differentiated, stable cell type. p75 transfected into human neuroblastoma cells (negative for p75) induced high-affinity binding of NGF and cell differentiation in response to NGF (Matsushima and Bogenmann, 1990). We determined that the ratio of NGF receptors changed from high trkA/low p75 to low trkA/high p75, the same profile present in C81-61 non-metastatic melanoma cells (Fig. 5A). Human melanocytes express p75 and experiments have shown that NGF stimulation modulates melanocyte gene expression (Yaar et al., 1991). Together, this suggests future studies examine trkA and its relative expression to p75 in metastatic melanoma.

Our ability to modulate p75 receptor levels by NGF exposure on a subset of metastatic melanoma cells that also renders the cells similar to non-metastatic cells and inhibits their ability to invade the embryonic neural crest microenvironment, suggests that we are selectively de-differentiating the stem cell population. The low-affinity NGF receptor, p75, also known as CD271, has been shown to be a marker of melanoma tumor stem cells (Boiko et al., 2010), a small subset of melanoma cells. When transplanted into mice, CD271-positive cells were the tumor-initiating population 90% of the time (Boiko et al., 2010). Although these cells were not assayed for their trkA expression, the overall vast majority of cells were tyrosinase and Mart-1 negative.

There are at least three possible explanations as to why our Mart-1:GFP-positive sorted cells lose expression over time. First, cells may maintain Mart-1:GFP expression, but the Mart-1:GFP reporter may lose its functionality. Second, a Mart-1:GFP-negative subpopulation is sustained through the FACS process. Lastly, there is a subpopulation of cells primed by NGF to re-express Mart-1:GFP and de-differentiate, however the conditions (for example, concentration of NGF) are not enough to drive a stable transdifferentiation and the cells revert back to a metastatic phenotype. Mart-1:GFP-negative cells proliferate roughly 2.5 times faster than Mart-1:GFP-positive cells (Fig. 4). Using a simple mathematical calculation based on number of Mart-1:GFP-positive and Mart-1:GFP-negative cells post-sorting (Fig. 4F), and analysis after each round of sorting (Fig. 4F), we would not expect the drastic decrease in Mart-1:GFP-positive cells observed 2-3 days post sort by the small population. Rather, a more rapid proliferation rate of Mart-1:GFP-negative cells (data not shown). On the contrary, if cells retained Mart-1:GFP expression but lost the reporter signal, we should see these cells proliferate at the same rate as GFP-positive cells and maintain equal ratios of these populations over time. Thus, these results indicate the likelihood of a combination of factors leading to the decrease in Mart-1:GFP-positive cells after selective sorting of this population.

Immunotherapeutic approaches have been investigated using T cells to target melanoma-associated antigens (MAA – Mart-1, tyrosinase, gp100) (Kawakami et al., 1994; Brichard et al., 1996). However, their success has been limited due to the heterogeneity (and lack) of expression of MAA in patient samples, leading to tumor cell escape from cytotoxic T lymphocytes. Surprisingly, the literature shows up to 40% of patient-derived primary tissue samples are Mart-1-negative (Cormier et al., 1998; Marincola et al., 1996), and up to 30% of melanoma cell lines (Marincola et al., 1996; de Vries et al., 1997). Given our findings that NGF has the ability to force Mart-1 re-expression, this lends the possibility of therapeutic approaches to modulating Mart-1 expression on metastatic melanoma cells, rendering them recognizable by cytotoxic T lymphocytes.

In summary, we have gained new insights into the potential of the embryonic neural crest microenvironment to reprogram phenotypically plastic metastatic melanoma cells towards a benign cell type. The discovery of NGF as a signal that underlies the reprogramming process may yield novel strategies to treat aggressive melanoma. For example, given the established focal therapy of implanting radioactive seeds into the prostate gland (Perera et al., 2016), the use of ultrasound guidance may be used to deliver NGF beads into the melanoma microenvironment to drive tumor cell differentiation. Tumor cell transplantations onto the vascularized chick chorioallentoic membrane (CAM) and subsequent analysis of metastasizing human tumor cells (as developed and validated in Bailey and Kulesa, 2014) offer a means to begin to test the efficacy of this strategy. Targeting NGF signaling as a differentiation strategy may be used to compliment traditional chemotherapies that target proliferation and angiogenesis. Although the chick embryo transplant model and CAM metastasis assay do not entirely mimic real-life human melanoma disease, further testing of NGF and other developmental signals that impact cell survival and differentiation across a broader set of metastatic melanoma tissues will help to determine the significance of this strategy.

## MATERIALS AND METHODS

### Chick embryos and cell culture

Fertilized White Leghorn chicken eggs (Ozark Hatchery, Meosho, MO, USA) were placed in a rocking incubator at 37°C (Kuhl, Flemington, NJ, USA) until appropriate age of development. After incubation embryos were staged according to the criteria of Hamburger and Hamilton (1951), and embryos were selected that were healthy and developing normally. The adult human metastatic cutaneous melanoma cell line C8161 and its poorly aggressive isogenic counterpart, C81-61, were isolated from an abdominal wall metastasis (Welch et al., 1991) and maintained as previously described (Hendrix et al., 2002). All cultures were determined to be free of mycoplasma contamination using a polymerase chain reaction-based detection system (Roche, Indianapolis, IN, USA). For certain experiments, tumor cell drops were generated by trypsinizing the cells (as for passaging) and placing 25 μl drops of resuspended c8161 cells on the inside surface of a 60×15 mm petri dish. 3 ml of media was placed in the bottom of the petri dish and the lid was then replaced, creating hanging drops of melanoma cells which were incubated for 24-48 h in a 37°C incubator supplied with 5% CO_2_. For cell proliferation experiments, 10 μl of 1 mM BrdU solution (Sigma-Aldrich, St. Louis, MO, USA) was added to cells in culture for 30 min, followed by fixation in 4°C and processed as below.

### Tumor cell transplantations

Chick embryos, 9-10 somites, were prepared for transplantation of melanoma cells by cutting a hole in the vitelline membrane above the neural tube using a sharpened tungsten needle. Hanging drops of C8161 cells (as cultured above) were cut into 100 μm×50 μm (wide) ×50 μm (thick) blocks using a sharpened tungsten needle. The melanoma cell block was then guided into the incision using the glass needle and gently tucked into the neural tube.

### Mart-1 reporter generation and testing

We obtained the Mart-1 reporter plasmid from Michihiro Konna (Nagoya University, Japan) and subcloned the reporter region into the lentiviral plasmid pLenti to make pLenti:h2b-mCherry/Mart1-EGFP. We then infected both C8161 and C81-61 cells. Since h2b-mCherry is expressed regardless of Mart-1 activation, we sorted cells based on mCherry expression to produce a pooled mix of stable integrants (polyclonal) expressing the Mart-1:GFP reporter. To confirm Mart-1 function in c8161 cells, we cultured the cells in 100 μM of all-trans retinoic acid (Sigma-Aldrich, St. Louis, MO, USA) for 48 h and confirmed GFP expression.

### Transwell migration assay

Cell were trysinized and seeded into the transwell migration insert (Corning, Corning, NY, USA) at 1×10^5^ per well (12-well plate format) and media with and without factor at 1 ml/well. Factors tested were NGF (Peprotech, Rocky Hill, NJ, USA) at 100 and 200 ng/ml and SDF (Peprotech, Rocky Hill, NJ, USA) at 100 ng/ml. Briefly, cells were allowed to invade across the membrane for 24 h. A Magellan plate reader (Tecan, Austria, GmbH) was used to determine the relative fluoresce units (RFU) of the cells that had crossed the membrane.

### *In vitro* migration assay

For *in vitro* cultures, glass bottom dishes (P35G-1.5-20-C, MatTek Corporation, Ashland, MA, USA) were coated for 30 min with 1 mg/ml of poly-l-lysine (Sigma-Aldrich), removed and dried for 30 min. The plates were then coated with 1 mg/ml of fibronectin (Sigma-Aldrich) for 30 min followed by 30 min of drying time. During this time, heparin-acrylic beads were washed multiple times in PBS and soaked in either 100 ng/ml of NGF, BDNF, SDF, NT3 (Peprotech, Rocky Hill, NJ, USA) or PBS (control). Hanging drops of c8161 cells (as cultured and cut above) were placed onto the coated glass-bottom dishes, in a minimum of media to maximize tissue adherence for 10 min at 37°C. During this time, fresh 2 mg/ml of collagen (BD Biosciences, San Jose, CA, USA) was prepared, and beads washed in PBS. Beads were positioned adjacent to the adhered neural tubes and 150 μl of collagen was placed over the cultures. If necessary, beads were pushed back into place before the collagen set. The collagen was allowed to set for 10-20 min at 37°C, and then 1.5 ml of DMEM +10%FBS was added and the cultures were returned to 37°C.

### Co-culture experiments

C8161 cells were plated on 8-well chamber slides (Sigma-Aldrich). For embryonic tissue co-culture experiments, embryos were incubated as above, removed from the egg and tissue regions of interest were excised using a sharpened tungsten needle [*n*=2 tissue pieces used per well for E1.5 cranial or trunk neural tube (NT) and E2.5 branchial arch 2 (ba2); *n*=2 2 tissue pieces for E2.5 and E3.5 trunk-using a 4 somite section between somites 18-24; *n*=5 DRG pieces for E3.5 DRG]. For co-cultures with specific factors, the following were used: NGF, BDNF, CXCL12, NT3, BMP4, BMP7 and FGF8 (all at 200 ng/ml; from Peprotech, Rocky Hill, NJ, USA). Slides were then incubated as above.

### Isolation of Mart-1 c8161 cells using FACS

Cells were trypsinized (Sigma-Aldrich) and centrifuged at 1000 rpm (445 ***g***) for 5 min, and resuspended in PBS with 2% FBS. To visualize viability, the resulting single cell suspension was stained with 2 μg/ml of 7AAD, and sorted using a MoFlo cytometer (Cytomation, Inx, Fort Collins, CO, USA). Mart-1:GFP-positive and Mart-1:GFP-negative populations were isolated.

### Gene profiling

Cell cultures were lysed, cDNA synthesized and pre-amplified using ABI Cells-to-Ct kit and Taqman Gene Expression assays as previously described in Bailey et al. (2012). RT-qPCR reactions were run on an ABI 7900 HT.

### Immunohistochemistry

Cultured cells were fixed in 4% PFA for 20 min. Immunocytochemistry on fixed cells were processed as in Rifkin et al. (2000) and Kasemeier-Kulesa et al. (2006). Primary antibodies included: Mart-1 (Cat #CM077, 1:100, Biocare Medical, Pacheco, CA, USA; Kulesa et al., 2006), trkA (Cat #2510, 1:300, Cell Signaling Technologies, Danvers, MA, USA; Huang and Reichardt, 2003), p75 (Cat#AB1554, 1:300, Millipore, Billerica, MA, USA) and BrdU (Cat#sc-70443, 1:200, Santa Cruz Biotechnology, Dallas, TX, USA; Roh et al., 2012).

### 3D confocal and time-lapse imaging

3D image z-stacks were collected on an inverted laser scanning confocal microscope (LSM5 Pascal, Carl Zeiss, Thornwood, NY, USA) using either a Plan-Neofluar 10×/0.3, Plan-Neofluar 40×/0.75 or C-Apochromat 40×/1.2 W objective (Carl Zeiss, Thornwood, NY, USA). The EGFP reporter was excited with the 488 nm laser line using the FITC filter and all other imaging parameters were as described in Kasemeier-Kulesa et al. (2005). Images were collected, processed and analyzed using AIM software (Carl Zeiss, Thornwood, NY, USA). Statistical analysis was performed using the Student's *t*-test.

## Supplementary Material

Supplementary information
