# Supplementary material for: NGF reprograms metastatic melanoma to a bipotent glial-melanocyte neural crest-like precursor
Source: Biol Open. 2017 Nov 24;7(1):bio030817. doi: 10.1242/bio.030817 (PMC5829509; doi:10.1242/bio.030817)
Supplement: Supplementary information [file biolopen-7-030817-s1.pdf]

© 2018. Published by The Company of Biologists Ltd | Biology Open (2018): doi:10.1242/bio.030817: Supplementary information

FIRST PERSON

First person – Jennifer Kasemeier-Kulesa

First Person is a series of interviews with the first authors of a selection of papers published in Biology Open, helping early-career researchers promote themselves alongside their papers. Jennifer Kasemeier-Kulesa is first author on 'NGF reprograms metastatic melanoma to a bipotent glial-melanocyte neural crest-like precursor', published in BiO. Jennifer is a Senior Research Specialist in the lab of Paul Kulesa in the Stowers Institute for Medical Research, USA, investigating mechanisms of cell migration in development and disease.

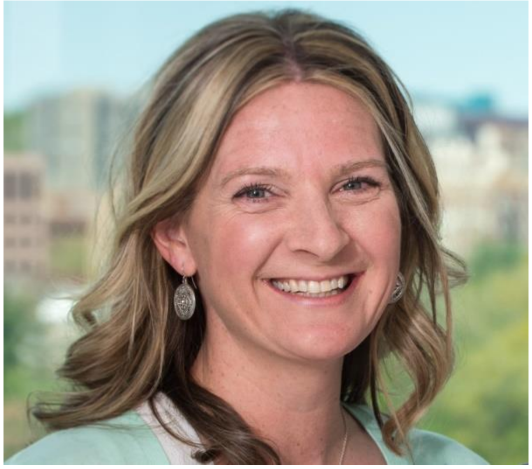

Jennifer Kasemeier-Kulesa

What is your scientific background and the general focus of your lab?

I started my scientific career as an organic chemist (St Martin's University, Olympia, WA), with an interest in learning about the basic mechanisms underlying developmental biology. These interests led me to a multi-disciplinary graduate program (IGERT-NIH funded) in chemistry and biology at Montana State University (advisor Frances Lefcort) where I obtained my PhD in Developmental Neuroscience. My PhD work uncovered unique cell migratory behaviors and signaling pathways of neural crest cells as they form autonomic ganglia of the peripheral nervous system. I have utilized this broad interdisciplinary training to further examine the mechanistic basis of neural crest migration and the pathogenesis of neural crest-derived cancers (melanoma and neuroblastoma); our laboratory is focused on working at the interface of development and disease. By understanding the regimented and controlled migration neural crest cells must abide by in the embryo to generate proper functioning organs and tissues, we can apply this knowledge to neural crest-derived cancers to understand uncontrolled migration and invasion in adult tissues.

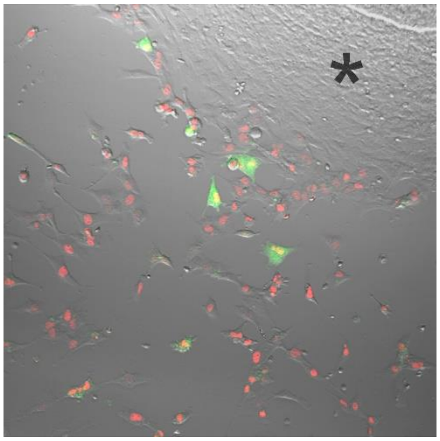

A subpopulation of C8161 metastatic melanoma cells (red) in the presence of chick trunk tissue (upper right, asterisk) are capable of re-expressing a melanocyte differentiation marker, Mart-1 (green).

How would you explain the main findings of your paper to non-scientific family and friends?

Melanoma cancer cells and their embryonic cell type of origin, pigment cells within the developing human embryo are ancestrally linked. We have found that when aggressive human melanoma cells are placed into the microenvironment of the early chick embryo (which is accessible to our manipulation and dynamic *in vivo* imaging) from which they were 'born', they revert behavior to that of their juvenile self and obey cues and characteristic behaviors of their embryonic neural crest cell neighbors. That is, the tumor cells behave themselves by not reforming tumors and migrate to proper tissues rather than invade everywhere. Further, we show that this new microenvironment they are in is able to influence a subset of these melanoma cells to stop 'misbehaving' like cancer cells and revert to a normal developing cell in the embryo.

"The robustness in the program of cells in general is amazing and the fact that we can potentially reprogram a cell that has 'gone astray' from its normal planned path is exciting!"

What are the potential implications of these results for your field of research?

The results of our study have a twofold implication to cancer research. First, our chick embryo transplantation model represents a rapid means to assess whether a neural crest-derived cancer cell is reprogrammable towards a less aggressive phenotype. That is, we envision our methods may rapidly screen human patient-derived melanoma cells to determine their susceptibility to respond to embryonic signals that typically drive multipotent embryonic cells to a differentiated cell type. Second, our discovery of nerve growth factor signaling as a reprogramming agent suggests that this signaling pathway within metastatic melanoma is a molecular inroad to further pursue as a potential therapeutic avenue.

Jennifer Kasemeier-Kulesa's contact details: Stowers Institute for Medical Research, Kansas City, USA.

E-mail: jck@stowers.org

What has surprised you the most while conducting your research?

General mechanisms of cells are surprisingly conserved, be it cells from human, chick, mouse or from the brain, skin or cartilage. Adult human metastatic cancer cells can be influenced by embryonic chick cells and potentially vice versa. The robustness in the program of cells in general is amazing and the fact that we can potentially reprogram a cell that has 'gone astray' from its normal planned path is exciting!

What, in your opinion, are some of the greatest achievements in your field and how has this influenced your research?

Rita Levi-Montalcini's pioneering work in placing tumor cells into the chick embryo to find massive nerve fiber growth towards these tumors led to her discovery of nerve growth factor (NGF). Personally, her unique circumstances of setting up a lab in her bedroom during World War II to continue her research, is a powerful reminder that no matter what technology you have available, science really comes down to your interest and dedication in a topic and being resourceful with what you have available.

What changes do you think could improve the professional lives of early-career scientists?

The Stowers Institute has developed several avenues to promote the research and development of young scientists through weekly science clubs that encourage students to present their work to an Institute-wide audience and annual young investigator research day. Also, professional societies such as the SDB and the AAA particularly encourage the participation and presentation of research by young scientists. So, further development of these types of programs continue to enhance the enthusiasm of early-career scientists.

What's next for you?

I am excited to follow up on this interesting population of metastatic melanoma cells, capable of being reprogrammed by NGF in the neural crest microenvironment. We think there is an important balance between NGF receptor expression that primes these cells for manipulation and the potential to alter cell plasticity.

Reference

Kasemeier-Kulesa, J. C., Romine, M. H., Morrison, J. A., Bailey, C. M., Welch, D. R. and Kulesa, P. M. (2018). NGF reprograms metastatic melanoma to a bipotent glial-melanocyte neural crest-like precursor. *Biol. Open* **7**, doi:10.1242/bio.030817.
